# The effect of dienogest and gonadotropin-releasing hormone agonist on pelvic pain after laparoscopic surgery for endometriosis: An RCT

**DOI:** 10.18502/ijrm.v22i12.18065

**Published:** 2025-01-31

**Authors:** Fatemeh Davari Tanha, Azam Rasti, Hamideh Pakniat, Shohreh Salimi Setudeh

**Affiliations:** ^1^Department of Obstetrics and Gynecology, Yas Hospital, Tehran University of Medical Sciences, Tehran, Iran.; ^2^Non-Communicable Diseases Research Center, Research Institute for Prevention of Non-Communicable Diseases, Qazvin University of Medical Sciences, Qazvin, Iran.

**Keywords:** Endometriosis, Gonadotropin-releasing hormone, Dienogest, Pelvic pain.

## Abstract

**Background:**

Endometriosis is a chronic inflammatory condition associated with debilitating chronic pelvic pain that affects women's quality of life. Several drugs have been used to reduce pain and psychological distress associated with this disease. Currently, gonadotropin-releasing hormone (GnRH) agonists and dienogest are the most widely used medical therapies for endometriosis.

**Objective:**

This study aimed to investigate the efficacy of dienogest and GnRH agonists in improving pelvic pain after laparoscopic surgery for endometriosis.

**Materials and Methods:**

In this randomized clinical trial study, 104 women with endometriosis who were referred to the Department of Reproductive Medicine of Yas hospital, Tehran, Iran, between April 2022 and March 2023 were studied. After laparoscopic surgery, individuals were randomly assigned into 2 groups (n = 52/each): the dienogest-administered group and the GnRH agonist-administered group. Participants were followed up at 3 months and pelvic pain was measured using the visual analog scale. Pelvic pain and adverse effects of drugs were compared between the groups.

**Results:**

Pelvic pain significantly improved in both treatment groups (p < 0.0001). No significant difference was observed in hot flashes and joint pain between the dienogest and GnRH agonist groups. However, a significant difference was found in vaginal dryness (p = 0.03) and decreased libido (p = 0.02). GnRH agonists and dienogest reduced irregular vaginal bleeding.

**Conclusion:**

Our results suggested that the effect of GnRH agonists and dienogest in improving pelvic pain for endometriosis is the same after a 3-month treatment period. However, these 2 drugs caused different adverse effects.

## 1. Introduction

Endometriosis is a chronic inflammatory disease caused by the abnormal presence of endometrial tissue outside the uterus cavity. It is a long-term, recurring, and debilitating condition that affects approximately 2–10% of women of reproductive age and 5–21% of women with severe pelvic pain (1, 2). The clinical manifestations of endometriosis can be very different, and some women with endometriosis are asymptomatic. However, they often experience a variety of painful symptoms including dysmenorrhea, dyspareunia, and chronic fatigue. Endometriosis, persistent pelvic pain, and fertility problems can cause subsequent psychological disorders such as anxiety and depression (3, 4).

The main goal of therapy attempts to reduce pain and long-term endometriosis-associated complications, such as infertility, fibrosis, adhesion, and malignancy. Effective treatment can improve the quality of life in women suffering from endometriosis and, as a result, decrease psychological problems (3, 5, 6). Although surgery for endometriosis can improve pain, medical therapy is necessary to prevent the recurrence of pain symptoms after surgery. Medications range from pain relief drugs to hormonal treatments, such as progestins, combined oral contraceptive pills, nonsteroidal anti-inflammatory drugs, gonadotropin-releasing hormone (GnRH) agonists, GnRH antagonists, aromatase inhibitors, danazol, gestrinone, selective estrogen receptor modulators. The appropriate medical treatment was chosen according to the individual's condition and the purpose of treatment. Different symptoms and complications of endometriosis, along with different treatment protocols, have led to only a 7% agreement between distinct guidelines for the treatment of this disease (7, 8). For this reason, drugs are constantly being investigated for their effects on various aspects of this disease, as well as their side effects.

GnRH agonists are commonly used to reduce pain in cases with endometriosis. These agonists effectively suppress endogenous gonadotropin secretion, lead to a hypoestrogenic state, induce medical menopause, and reduce ovarian steroidogenesis by suppressing ovulation. However, the hypoestrogenic side effects, including decreased bone mineral density have limited long-term treatment (9–11). In comparison, dienogest causes a moderate decrease in estrogen levels, which leads to the detachment of endometrial tissue and atrophy of endometriosis lesions. This tolerable reduction in estrogen levels can cause fewer side effects (12, 13).

In this study, we investigated and compared the therapeutic effects of GnRH agonists and dienogest drugs on various aspects of this disease as well as their adverse effects.

## 2. Materials and Methods

### Study design and participants

This parallel randomized controlled trial was conducted on infertile women who referred to the Department of Reproductive Medicine of Yas hospital, Tehran, Iran, between April 2022 and March 2023. 200 infertile women with clinically suspected endometriosis were enrolled. Participants underwent clinical examination, transvaginal ultrasound, and pelvic magnetic resonance imaging. Cases with deep infiltrating endometriosis (grade 3 and 4 endometriosis) and those aged between 20 and 42 yr, were included in this research. Those with hypersensitivity to dienogest or GnRH agonist were excluded.

### Sample size

The sample size in this research was determined to be 52 in each group by considering the power of 80% with a difference of 22.4 ± 11.85 (dienogest therapy) and 16.15 ± 10.69 (GnRH-a therapy) in the mean visual analog scale (VAS) score based on a previous research (14), and used the following formula at alpha = 0.05: 


$$n=\frac{{\left(Z_{1-\frac{\alpha }{2}}+Z_{1-\beta }\right)}^{2}\left(\sigma_{1}^{2}+\sigma_{2}^{2}\right)}{{\left(\mu_{1}-\mu_{2}\right)}^{2}}$$

### Implementation

The infertility fellowship assistant randomly allocated and assigned participants to interventions. The personnel of the Department of Reproductive Medicine of Yas hospital, Tehran, Iran executed the registration of participants.

### Randomization

Random allocation rules were used. First, letters A and B were written on special papers that were not marked inside. They were then placed in a bag for each woman, after obtaining informed consent. A paper was removed randomly without replacement, and based on the letter written on it, the desired intervention was performed for the woman. In addition, letter A was considered for dienogest intervention, and letter B was for GnRH agonist intervention.

### Interventions

To treat infertility, ovulation stimulation was performed, and the embryos were frozen. Then laparoscopic surgery was done, and the participants were randomly divided into 2 groups. In the first group, dienogest 2 mg tablets (Atipharmed, Iran) were prescribed daily for 3 months continuously. In the other group, GnRH agonist 11.25 mg (Diphereline, Ipsen, France) was administered only once after surgery.

### Outcomes and data collection

The primary outcome for this study was the pelvic pain intensity, including dysmenorrhea, dyspareunia, and dyschezia that was measured using the 10 cm VAS-scale, ranging from 0 (no pain) to 10 (very severe pain). Before laparoscopic surgery, the pelvic pain intensity of the participants was measured using the female sexual function index questionnaire and VAS, and after laparoscopic surgery and treatment with dienogest and GnRH agonist, individuals pain was re-evaluated and compared with the preoperative values. Other parameters evaluated in this study included the adverse effects of both drugs, such as hot flashes, joint pain, vaginal dryness, decreased libido, irregular vaginal bleeding, and hirsutism, which were measured by interviewing and completing a questionnaire.

### Ethical Considerations

The research protocol was carried out according to international laws and after approval by the Ethics Committee of the Tehran University of Medical Sciences, Tehran, Iran (Code: IR.TUMS.MEDECINE.REC.1400.964), has been registered in the Iranian Registry of Clinical Trials (Code: IRCT20091012002576N25, registration date: March 03, 2022, last update: August 21, 2024). In this study, the participants were explained about the methods before entering the research, and all the intervention steps were completed after obtaining written informed consent. The research organizers ensured there would be no disturbances in the usual treatment of participants. Ethical principles related to the confidentiality of participants information were also considered.

### Statistical Analysis

All statistical analysis was performed using Statistical Package for the Social Sciences software (SPSS, version 22.0 for Windows; SPSS Inc., Chicago, Illinois, USA) and GraphPad Prism8. The normal distribution of the data was assessed by the Kolmogorov-Smirnov test. Nonparametric variables were compared using the Wilcoxon signed-rank test or Mann-Whitney test, *t* test was applied for comparing parametric variables, and Fisher's exact test for categorical variables. The significance level was set at p < 0.05.

## 3. Results

**Figure 1:**
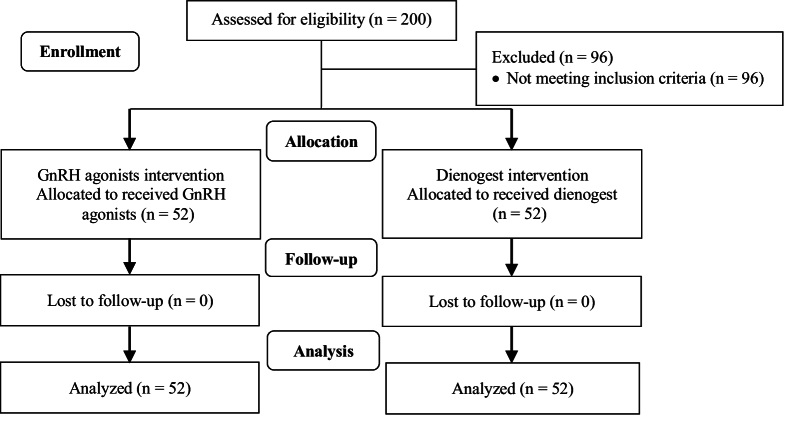
Trial design flowchart. GnRH: Gonadotropin-releasing hormone.

As was shown in figure 1, in this clinical trial, 200 cases were evaluated for eligibility, of which 96 cases did not meet the inclusion criteria and were excluded. 104 participants were assigned to the intervention, of which 52 women used dienogest and 52 women used GnRH agonists. Demographic characteristics of the study subjects are shown in table I.

The efficacy of the 2 drugs in improving endometriosis-related complications was investigated in both groups. We found that both dienogest and GnRH agonists were effective treatments in improving dysmenorrhea, dyspareunia, and dyschezia (p < 0.0001) (Table II).

The use of GnRH agonists and dienogest improved irregular vaginal bleeding including menorrhagia, metrorrhagia, and oligomenorrhea (p = 0.0003, p = 0.0007). In the present study, these drugs did not cause menorrhagia, metrorrhagia, and oligomenorrhea (Table II).

On examining the adverse effects caused by these drugs, the 2 groups did not differ in terms of the number of women with hot flashes or joint pain. A significant difference was observed in vaginal dryness and loss of libido between the 2 groups. Vaginal dryness was more frequently observed in GnRH agonists users (p = 0.03), and decreased libido was found in dienogest users (p = 0.02). None of the individuals in the 2 groups had hirsutism (Table III).

**Table 1:** Participants baseline characteristics

**Demographic characteristics**		**Groups**		**P-value**
		**Dienogest (n = 52)**	**GnRH agonist (n = 52)**	
BMI (kg/m^2^)*		24.90 (28.36–23.11)	23.59 (26.53–19.95)	0.14
Age (yr)**		35.00 (38–32)	32.00 (35–30)	0.06
Endometrioma localization***				
	**Bilateral**	5 (10.8)	11 (22.4)	0.0649
	**Right unilateral**	7 (15.2)	2 (4)	
	**Left unilateral**	4 (8.6)	7 (14.2)	
USL involvement***				
	**Yes**	29 (55.7)	38 (73.07)	0.0653
	**No**	23 (44.2)	14 (26.92)	
Rectovaginal septal endometriosis***				
	**Yes**	27 (51.9)	30 (58.8)	0.5539
	**No**	25 (48)	21 (41.1)	
*Data presented as median (IQR), *t* test. **Data presented as median (IQR), Mann–Whitney test. ***Data presented as n (%), Chi-square test. GnRH: Gonadotropin-releasing hormone agonist, BMI: Body mass index, USL: Uterosacral ligament				

**Table 2:** The effects of dienogest and GnRH agonist on different aspects of endometriosis before and after treatment

**Variables**	**Dienogest**			**GnRH agonist**		
	**Before**	**After**	**P-value**	**Before**	**After**	**P-value**
Dysmenorrhea*	7.981 ± 2.356	3.654 ± 2.457	< 0.0001	8.115 ± 1.916	3.442 ± 2.461	< 0.0001
Dyspareunia**	5.500 (3.250–8.000)	2.000 (0.000–4.000)	< 0.0001	5.000 (3.000–7.000)	0.000 (0.000–4.000)	< 0.0001
Dyschezia**	3.000 (0.000–7.000)	2.000 (0.000–4.000)	< 0.0001	3.000 (0.000–8.000)	2.000 (0.000–6.000)	< 0.0001
**Irregular vaginal** bleeding***	22 (42.31)	6 (11.54)	0.0007	20 (38.46)	4 (7.69)	0.0003
*Data presented as Mean ± SD, Wilcoxon matched-pairs signed rank test. **Data presented as Median (IQR), Wilcoxon matched-pairs signed rank test. ***Data presented as n (%), Fisher's exact test. GnRH: Gonadotropin-releasing hormone agonist						

**Table 3:** Adverse effects observed in the dienogest group compared with the GnRH agonist group

**Variables**	**Dienogest (n = 52)**	**GnRH agonist (n = 52)**	**P-value**	**Relative risk**	**95% CI**
**Hot flush**	16 (30.77)	18 (34.62)	0.83	0.8889	0.5114–1.537
**Vaginal dryness**	13 (25.00)	24 (46.15)	0.03	0.5417	0.3082–0.9260
**Joint pain**	10 (19.23)	15 (28.85)	0.35	0.6667	0.3317–1.319
**Decreased libido**	24 (46.15)	12 (23.08)	0.02	2.000	1.148–3.592
**Hirsutism**	0 (0)	0 (0)	-	-	-
Data are presented as n (%), Fisher's exact test. GnRH: Gonadotropin-releasing hormone agonist					

## 4. Discussion

In this study, the efficacy and adverse effects of GnRH agonist and dienogest were investigated during a 3-month treatment period in women with endometriosis. The efficacy of both drugs was the same in reducing dysmenorrhea, dyspareunia, and dyschezia, and the 2 drugs did not differ in terms of the number of women with hot flashes and joint pain; however, vaginal dryness was more common in GnRH agonists and decreased libido in dienogest users. The use of GnRH agonists and dienogest reduced the amount of irregular vaginal bleeding and these drugs did not cause menorrhagia, metrorrhagia, and oligomenorrhea.

Endometriosis is a chronic disease that may require ongoing management across the life course. Although surgical intervention can help relieve pain and improve fertility, the risk of disease recurrence is high (40–50% at 5 yr) (15). These drugs can effectively delay surgery and reduce the risk of endometriosis recurrence after surgery.

Endometriosis is an estrogen-dependent disease; therefore, one of the mechanisms proposed to prevent the disease from progression is to reduce or stop estrogen production (7). GnRH agonists inhibit the secretion of follicle-stimulating hormone, prevent the production of estrogen, and produce a hypoestrogenic state (10). Dienogest is a 4 ${\;}^{\text{th}}$ -generation selective progestin, which combines the pharmacological properties of 19-nortestosterone and progesterone derivatives, offering a pronounced special effect on endometriotic lesions. Dienogest is associated with mild inhibition of the hypothalamic-pituitary-ovarian axis, moderate reduction in gonadotropin secretion, and estradiol production. Continuous administration of dienogest results in hypergestogenic and hypoestrogenic endocrine environment. Dienogest also demonstrates antiproliferative, anti-inflammatory, and anti-angiogenic effects that effectively reduce the growth of endometriosic lesions (15–17). Our study, like other studies in this field, showed that both dienogest and GnRH agonists were associated with a highly significant reduction of pelvic pain such as dysmenorrhea, dyschezia, and dyspareunia (7, 16).

The use of GnRH agonists and dienogest reduced the amount of irregular vaginal bleeding. GnRH agonists improved menorrhagia in all 11 affected women, oligomenorrhea in the only affected person, and metrorrhagia in 4 out of 8 affected women. Dienogest improved menorrhagia in 4 out of 5 affected women, oligomenorrhea in 3 out of 5 affected women, and metrorrhagia in 9 out of 12 affected women. Although some studies have pointed to the beneficial effects of GnRH agonists in the treatment of menorrhagia, other studies have reported that dienogest and GnRH agonists may cause irregular vaginal bleeding which progressively decreases with continued treatment (3, 4, 11, 18). In the present study, these drugs did not cause new cases of menorrhagia, metrorrhagia, and oligomenorrhea; moreover, they improved these problems.

Although GnRH agonists and dienogest can effectively relieve endometriosis-related pain, the treatment is associated with significant hypoestrogenic complications. The GnRH agonist-derived estrogen reduction resulted in hot flashes in more than 60% of women, bone loss in 20–60%, joint pain, vaginal dryness, and decreased libido (12, 13). Consequently, treatment with GnRH agonists is limited to 6 months in the absence of “add-back” therapy with steroids. On the other hand, studies have shown that the milder effects of dienogest on estrogen levels can reduce complications (11, 18). Several studies reported the rate of hot flashes caused by dienogest to be < 15% (15, 19, 20). In our study, the rate of hot flashes was 34.62% after 12 wk of GnRH agonists therapy and 30.77% after dienogest therapy, and no significant difference was observed between the 2 groups. The reason for the difference in the rate of hot flashes caused by dienogest in other studies may be due to the difference in the duration of administration because some of the adverse effects of drugs decrease with treatment. In studies where the duration of administration was 24 wk, 16 wk, and 52 wk, 11%, 9.6%, and 3.4% of women reported hot flashes, respectively (15, 16, 21).

Also, similar to our study, no significant difference was observed in joint pain between the dienogest and GnRH-agonist groups (16).

However, the 2 groups showed significant differences in the rates of vaginal dryness and decreased libido. Due to the anti-androgenic effects of dienogest, it does not cause acne, alopecia, weight gain, or hirsutism, but it leads to a decrease in libido (13, 22). Vaginal dryness occurs due to low levels of estrogen, and since GnRH agonist therapy is accompanied by more severe estrogen depletion compared to dienogest, as our results also showed, vaginal dryness is expected to be more common in GnRH agonist users (5, 23, 24). Therefore, in short-term treatments, each of these 2 drugs can be prescribed according to the individual's condition, and the access to drug. For example, GnRH agonists is suggested every 3 months for a short time for women who may forget to take dienogest daily, without worrying about its serious adverse effects.

According to the previous studies, the effectiveness and adverse effects of these 2 drugs differ during the treatment and are related to treatment duration (10, 25, 26). It is suggested that in future studies on long-term therapy, patients should be evaluated at specific time intervals to determine how much improvement and adverse effects are expected for each time interval.

## 5. Conclusion

It appears that the effectiveness of GnRH agonists and dienogest in a 3-month treatment for endometriosis is the same in improving dysmenorrhea, dyspareunia, and dyskinesia, while the adverse effects of these 2 drugs may be different. Therefore, while research continues to design more effective drugs, a comprehensive understanding of the effects of existing drugs can help manage drug prescriptions based on the disease and patient's condition.

##  Data Availability

The data supporting the findings of this study are available from the corresponding author, upon reasonable request.

##  Author Contributions

H. Pakniat and F. Davari Tanha designed the study and conducted the research. A. Rasti and Sh. Salimi Setudeh monitored, evaluated, and analyzed the results of the study. Further, A. Rasti, F. Davari Tanha, and H. Pakniat reviewed the article. All authors approved the final manuscript and take responsibility for the integrity of the data.

##  Conflict of Interest

The authors declared no potential conflict of interest regarding the research, authorship, and/or publication of this article.
